# Ethyl *N*-[4-(3-methyl-4,5-dihydro­benzo[*g*]indazol-1-yl)phenyl­sulfon­yl]thio­carbam­ate ethanol monosolvate

**DOI:** 10.1107/S1600536811034076

**Published:** 2011-08-27

**Authors:** Abdullah M. Asiri, Abdulrahman O. Al-Youbi, Hassan M. Faidallah, Seik Weng Ng, Edward R. T. Tiekink

**Affiliations:** a Chemistry Department, Faculty of Science, King Abdulaziz University, PO Box 80203, Jeddah,Saudi Arabia; b Center of Excellence for Advanced Materials Research, King Abdulaziz University, PO Box 80203, Jeddah, Saudi Arabia; c Department of Chemistry, University of Malaya, 50603 Kuala Lumpur, Malaysia

## Abstract

The title compound, C_21_H_20_N_3_O_3_S_2_·CH_3_CH_2_OH, comprises two independent organic mol­ecules and two ethanol solvent mol­ecules. The mol­ecules are related by pseudo-mirror symmetry. In both mol­ecules, the N-bound benzene ring is twisted out of the plane of the pyrazole ring [the dihedral angles are 51.4 (3) and 44.1 (3)°, respectively]. Similarly, the benzene ring of the 1,2-dihydro­naphthalene residue is inclined with respect to the five-membered ring [dihedral angles 18.3 (3) and 22.2 (3)°]. Overall, each mol­ecule has a flattened U shape. Dimeric aggregates mediated by O—H⋯N(pyrazole) and amide-N—H⋯O hydrogen bonds feature in the crystal packing, whereby the ethanol mol­ecules link the independent organic mol­ecules, leading to four-mol­ecule aggregates.

## Related literature

For background to the biological activity of species related to the title compound, see: Faidallah *et al.* (2007 ▶); Al-Saadi *et al.* (2008 ▶).
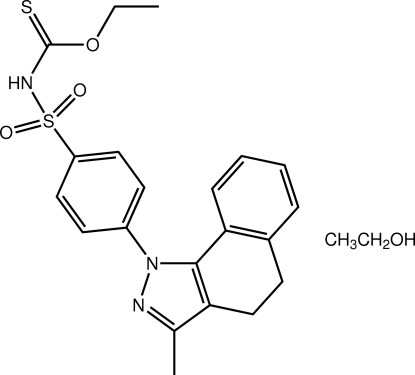


## Experimental

### 

#### Crystal data

C_21_H_21_N_3_O_3_S_2_·C_2_H_6_O
*M* *_r_* = 473.60
Monoclinic, 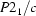

*a* = 22.673 (2) Å
*b* = 12.5563 (8) Å
*c* = 17.3831 (17) Å
β = 110.410 (11)°
*V* = 4638.1 (7) Å^3^
*Z* = 8
Mo *K*α radiation
μ = 0.27 mm^−1^
*T* = 100 K
0.25 × 0.25 × 0.05 mm

#### Data collection

Agilent SuperNova Dual diffractometer with Atlas detector
Absorption correction: multi-scan (*CrysAlis PRO*; Agilent, 2010 ▶) *T*_min_ = 0.786, *T*_max_ = 1.000
21133 measured reflections
10333 independent reflections
4871 reflections with *I* > 2σ(*I*)
*R*_int_ = 0.089

#### Refinement

*R*[*F* ^2^ > 2σ(*F* ^2^)] = 0.087
*wR*(*F* ^2^) = 0.261
*S* = 1.03
10333 reflections
581 parameters
H-atom parameters constrained
Δρ_max_ = 0.80 e Å^−3^
Δρ_min_ = −0.67 e Å^−3^

### 

Data collection: *CrysAlis PRO* (Agilent, 2010 ▶); cell refinement: *CrysAlis PRO*; data reduction: *CrysAlis PRO*; program(s) used to solve structure: *SHELXS97* (Sheldrick, 2008 ▶); program(s) used to refine structure: *SHELXL97* (Sheldrick, 2008 ▶); molecular graphics: *ORTEP-3* (Farrugia, 1997 ▶), *DIAMOND* (Brandenburg, 2006 ▶) and *Qmol* (Gans & Shalloway, 2001 ▶); software used to prepare material for publication: *publCIF* (Westrip, 2010 ▶).

## Supplementary Material

Crystal structure: contains datablock(s) global, I. DOI: 10.1107/S1600536811034076/pk2343sup1.cif

Structure factors: contains datablock(s) I. DOI: 10.1107/S1600536811034076/pk2343Isup2.hkl

Supplementary material file. DOI: 10.1107/S1600536811034076/pk2343Isup3.cml

Additional supplementary materials:  crystallographic information; 3D view; checkCIF report

## Figures and Tables

**Table 1:** Hydrogen-bond geometry (Å, °)

*D*—H⋯*A*	*D*—H	H⋯*A*	*D*⋯*A*	*D*—H⋯*A*
N3—H3⋯O8	0.88	1.82	2.700 (5)	174
N6—H6⋯O7	0.88	1.88	2.750 (6)	170
O7—H7⋯N1	0.84	2.03	2.839 (6)	161
O8—H8⋯N4	0.84	1.98	2.807 (5)	170

**Fig. 1.**
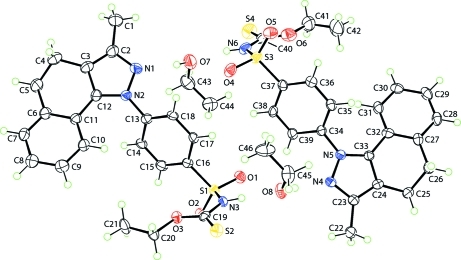
The molecular structures of the two independent organic molecules and two independent ethanol molecules that comprise the crystallographic asymmetric unit in (I) showing the atom-labelling scheme and displacement ellipsoids at the 50% probability level.

**Fig. 2.**
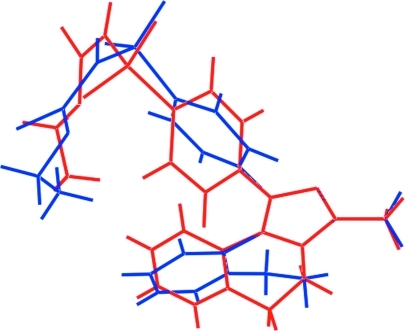
Overlay diagram of the two independent organic molecules. The image in red contains the S1 atom.

**Fig. 3.**
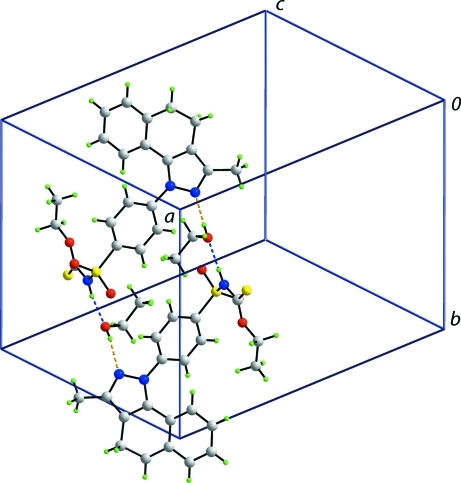
Four-molecule aggregate in (I) mediated by O—H···N and N—H···O hydrogen bonding, shown as orange and blue dashed lines, respectively.

## supplementary crystallographic information

### Comment

The structural analysis of the title compound, (I), was conducted in the context of on-going investigations into the biological activity of related species (Faidallah *et al.*, 2007; Al-Saadi *et al.*, 2008).

Two independent organic molecules and two solvent ethanol molecules comprise the crystallographic asymmetric unit in (I), Fig. 1. As seen from the overlay diagram, Fig. 2, the molecules are related by a pseudo mirror plane but there are measurable conformational differences between them. Focusing upon *N*-bound-benzene rings first, the dihedral angle formed between it and the pyrazole ring is 51.4 (3) and 44.1 (3)°, respectively for the S1- and S2-containing molecules. With respect to the 1,2-dihydronaphthalene residue, the β-methylene groups lie to opposite sides of the plane through the pyrazole ring in the two molecules but to approximately the same extent as reflected in the dihedral angles formed between the five-membered ring and the benzene rings of 18.3 (3) and 22.2 (3)°, respectively. Globally, the molecules have a flattened U-shape as the terminal ethoxycarbothioamide group is orientated to the same side of the molecule as the 1,2-dihydronaphthalene residue, with both directed over the *N*-bound benzene ring.

As anticipated there is hydrogen bonding in operation in the crystal structure of (I), Table 1. Thus, there are close associations between the ethanol and organic molecules with each solvent molecule forming a hydrogen bond to a pyrazole-N and at the same time accepting a hydrogen bond from an amide-H. As shown in Fig. 3, the ethanol molecules link the independent organic molecules into four-molecule aggregates.

### Experimental

A mixture of 3-methyl-2-(*p*-sulfamylphenyl)-4,5-dihydronaphtho[1,2-*c*]pyrazole (10 mmol) and anhydrous K_2_CO_3_ (20 mmol) in dry acetone (25 ml) was stirred and treated with phenylisothiocyanate (12 mmol). After the mixture was stirred and refluxed for 10 h, acetone was removed under pressure. The isolated solid mass dissolved in water and acidified with 2 N HCl. The crude product was purified by recrystallization from ethanol as colourless plates m.p. 457–458 K.

### Refinement

Carbon-bound H atoms were placed in calculated positions [O—H = 0.84 Å, N—H = 0.88 Å and C—H 0.95 to 0.99 Å, *U*_iso_(H) 1.2 to 1.5*U*_eq_(parent atom)] and were included in the refinement in the riding model approximation.

### Crystal data

**Table:**

C_21_H_21_N_3_O_3_S_2_·C_2_H_6_O	*F*(000) = 2000
*M**_r_* = 473.60	*D*_x_ = 1.356 Mg m^−^^3^
Monoclinic, *P*2_1_/*c*	Mo *K*α radiation, λ = 0.71073 Å
Hall symbol: -P 2ybc	Cell parameters from 2528 reflections
*a* = 22.673 (2) Å	θ = 2.3–29.2°
*b* = 12.5563 (8) Å	µ = 0.27 mm^−^^1^
*c* = 17.3831 (17) Å	*T* = 100 K
β = 110.410 (11)°	Plate, colourless
*V* = 4638.1 (7) Å^3^	0.25 × 0.25 × 0.05 mm
*Z* = 8	

### Data collection

**Table:**

Agilent SuperNova Dual diffractometer with Atlas detector	10333 independent reflections
Radiation source: SuperNova (Mo) X-ray Source	4871 reflections with *I* > 2σ(*I*)
mirror	*R*_int_ = 0.089
Detector resolution: 10.4041 pixels mm^-1^	θ_max_ = 27.5°, θ_min_ = 2.3°
ω scans	*h* = −29→29
Absorption correction: multi-scan (*CrysAlis PRO*; Agilent, 2010)	*k* = −16→16
*T*_min_ = 0.786, *T*_max_ = 1.000	*l* = −22→15
21133 measured reflections	

### Refinement

**Table:**

Refinement on *F*^2^	Primary atom site location: structure-invariant direct methods
Least-squares matrix: full	Secondary atom site location: difference Fourier map
*R*[*F*^2^ > 2σ(*F*^2^)] = 0.087	Hydrogen site location: inferred from neighbouring sites
*wR*(*F*^2^) = 0.261	H-atom parameters constrained
*S* = 1.03	*w* = 1/[σ^2^(*F*_o_^2^) + (0.0853*P*)^2^ + 2.6042*P*] where *P* = (*F*_o_^2^ + 2*F*_c_^2^)/3
10333 reflections	(Δ/σ)_max_ = 0.001
581 parameters	Δρ_max_ = 0.80 e Å^−^^3^
0 restraints	Δρ_min_ = −0.67 e Å^−^^3^

### Special details

**Table:**

Geometry. All e.s.d.'s (except the e.s.d. in the dihedral angle between two l.s. planes) are estimated using the full covariance matrix. The cell e.s.d.'s are taken into account individually in the estimation of e.s.d.'s in distances, angles and torsion angles; correlations between e.s.d.'s in cell parameters are only used when they are defined by crystal symmetry. An approximate (isotropic) treatment of cell e.s.d.'s is used for estimating e.s.d.'s involving l.s. planes.
Refinement. Refinement of *F*^2^ against ALL reflections. The weighted *R*-factor *wR* and goodness of fit *S* are based on *F*^2^, conventional *R*-factors *R* are based on *F*, with *F* set to zero for negative *F*^2^. The threshold expression of *F*^2^ > σ(*F*^2^) is used only for calculating *R*-factors(gt) *etc*. and is not relevant to the choice of reflections for refinement. *R*-factors based on *F*^2^ are statistically about twice as large as those based on *F*, and *R*-factors based on ALL data will be even larger.

### Fractional atomic coordinates and isotropic or equivalent isotropic displacement parameters (Å^2^)

**Table:**

	*x*	*y*	*z*	*U*_iso_*/*U*_eq_	
S1	0.63991 (6)	0.65962 (9)	0.32992 (7)	0.0230 (3)	
S2	0.48267 (6)	0.75296 (10)	0.39121 (8)	0.0304 (3)	
S3	0.85528 (6)	0.60975 (10)	0.68288 (8)	0.0286 (3)	
S4	1.00959 (7)	0.52174 (13)	0.61264 (11)	0.0488 (4)	
O1	0.67067 (16)	0.5629 (3)	0.3649 (2)	0.0299 (8)	
O2	0.61421 (15)	0.6701 (3)	0.24243 (19)	0.0269 (8)	
O3	0.55384 (15)	0.8286 (2)	0.3102 (2)	0.0258 (8)	
O4	0.82340 (15)	0.7052 (3)	0.6486 (2)	0.0354 (9)	
O5	0.87921 (16)	0.5965 (3)	0.7695 (2)	0.0359 (9)	
O6	0.94417 (16)	0.4448 (3)	0.7010 (2)	0.0425 (10)	
O7	0.9183 (2)	0.7617 (3)	0.5384 (2)	0.0476 (11)	
H7	0.9009	0.8209	0.5374	0.071*	
O8	0.57919 (17)	0.5072 (3)	0.4670 (2)	0.0327 (9)	
H8	0.5934	0.4454	0.4667	0.049*	
N1	0.88022 (19)	0.9783 (3)	0.5172 (3)	0.0329 (11)	
N2	0.82352 (18)	1.0079 (3)	0.4619 (2)	0.0250 (9)	
N3	0.58346 (16)	0.6722 (3)	0.3695 (2)	0.0220 (9)	
H3	0.5807	0.6215	0.4031	0.026*	
N4	0.61940 (17)	0.2944 (3)	0.4789 (2)	0.0228 (9)	
N5	0.67529 (18)	0.2618 (3)	0.5351 (2)	0.0228 (9)	
N6	0.91276 (18)	0.6036 (3)	0.6451 (3)	0.0313 (10)	
H6	0.9169	0.6585	0.6160	0.038*	
C1	0.9816 (3)	1.0621 (5)	0.5914 (4)	0.0582 (19)	
H1A	0.9936	0.9877	0.6054	0.087*	
H1B	0.9848	1.1017	0.6413	0.087*	
H1C	1.0097	1.0940	0.5659	0.087*	
C2	0.9157 (2)	1.0667 (4)	0.5330 (4)	0.0380 (14)	
C3	0.8815 (2)	1.1527 (4)	0.4871 (4)	0.0368 (14)	
C4	0.8972 (3)	1.2687 (5)	0.4828 (4)	0.0478 (16)	
H4A	0.9429	1.2774	0.4949	0.057*	
H4B	0.8853	1.3100	0.5238	0.057*	
C5	0.8603 (2)	1.3084 (5)	0.3963 (4)	0.0453 (16)	
H5A	0.8645	1.3868	0.3948	0.054*	
H5B	0.8789	1.2775	0.3575	0.054*	
C6	0.7913 (2)	1.2797 (4)	0.3684 (3)	0.0300 (12)	
C7	0.7462 (2)	1.3491 (4)	0.3195 (3)	0.0291 (12)	
H7A	0.7589	1.4156	0.3045	0.035*	
C8	0.6829 (2)	1.3228 (4)	0.2922 (3)	0.0322 (12)	
H8A	0.6525	1.3703	0.2576	0.039*	
C9	0.6642 (2)	1.2273 (4)	0.3156 (3)	0.0323 (13)	
H9	0.6207	1.2100	0.2983	0.039*	
C10	0.7083 (2)	1.1564 (4)	0.3640 (3)	0.0268 (11)	
H10	0.6950	1.0910	0.3799	0.032*	
C11	0.7721 (2)	1.1805 (4)	0.3896 (3)	0.0267 (11)	
C12	0.8226 (2)	1.1134 (4)	0.4426 (3)	0.0273 (11)	
C13	0.7777 (2)	0.9263 (4)	0.4294 (3)	0.0247 (11)	
C14	0.7501 (2)	0.9129 (4)	0.3456 (3)	0.0285 (12)	
H14	0.7609	0.9586	0.3091	0.034*	
C15	0.7067 (2)	0.8328 (4)	0.3154 (3)	0.0298 (12)	
H15	0.6875	0.8228	0.2579	0.036*	
C16	0.6911 (2)	0.7664 (4)	0.3699 (3)	0.0217 (10)	
C17	0.7199 (2)	0.7789 (4)	0.4539 (3)	0.0232 (11)	
H17	0.7103	0.7316	0.4905	0.028*	
C18	0.7627 (2)	0.8606 (4)	0.4841 (3)	0.0247 (11)	
H18	0.7817	0.8715	0.5416	0.030*	
C19	0.5410 (2)	0.7528 (4)	0.3555 (3)	0.0239 (11)	
C20	0.5116 (2)	0.9195 (4)	0.2846 (3)	0.0274 (11)	
H20A	0.5040	0.9519	0.3323	0.033*	
H20B	0.4707	0.8977	0.2436	0.033*	
C21	0.5449 (2)	0.9962 (4)	0.2478 (4)	0.0382 (14)	
H21A	0.5191	1.0603	0.2297	0.057*	
H21B	0.5518	0.9628	0.2007	0.057*	
H21C	0.5855	1.0157	0.2890	0.057*	
C22	0.5185 (2)	0.2126 (4)	0.3980 (3)	0.0302 (12)	
H22A	0.5066	0.2873	0.3854	0.045*	
H22B	0.4893	0.1788	0.4209	0.045*	
H22C	0.5168	0.1754	0.3477	0.045*	
C23	0.5837 (2)	0.2068 (4)	0.4591 (3)	0.0225 (10)	
C24	0.6165 (2)	0.1188 (4)	0.5031 (3)	0.0216 (10)	
C25	0.5996 (2)	0.0043 (4)	0.5062 (3)	0.0273 (12)	
H25A	0.6133	−0.0379	0.4673	0.033*	
H25B	0.5534	−0.0034	0.4907	0.033*	
C26	0.6328 (2)	−0.0357 (4)	0.5943 (3)	0.0287 (12)	
H26A	0.6133	−0.0018	0.6310	0.034*	
H26B	0.6267	−0.1137	0.5960	0.034*	
C27	0.7019 (2)	−0.0116 (4)	0.6251 (3)	0.0249 (11)	
C28	0.7457 (2)	−0.0822 (4)	0.6748 (3)	0.0300 (12)	
H28	0.7316	−0.1468	0.6911	0.036*	
C29	0.8099 (3)	−0.0602 (4)	0.7014 (3)	0.0338 (13)	
H29	0.8393	−0.1096	0.7353	0.041*	
C30	0.8310 (2)	0.0348 (4)	0.6778 (3)	0.0296 (12)	
H30	0.8747	0.0500	0.6951	0.035*	
C31	0.7880 (2)	0.1061 (4)	0.6294 (3)	0.0288 (12)	
H31	0.8024	0.1705	0.6132	0.035*	
C32	0.7233 (2)	0.0856 (4)	0.6036 (3)	0.0251 (11)	
C33	0.6746 (2)	0.1558 (4)	0.5512 (3)	0.0217 (10)	
C34	0.7211 (2)	0.3412 (4)	0.5721 (3)	0.0215 (10)	
C35	0.7516 (2)	0.3434 (4)	0.6567 (3)	0.0243 (11)	
H35	0.7427	0.2914	0.6908	0.029*	
C36	0.7954 (2)	0.4234 (4)	0.6902 (3)	0.0245 (11)	
H36	0.8181	0.4248	0.7476	0.029*	
C37	0.8058 (2)	0.5001 (4)	0.6406 (3)	0.0225 (11)	
C38	0.7746 (2)	0.4989 (4)	0.5558 (3)	0.0251 (11)	
H38	0.7821	0.5532	0.5222	0.030*	
C39	0.7328 (2)	0.4180 (4)	0.5214 (3)	0.0243 (11)	
H39	0.7120	0.4146	0.4636	0.029*	
C40	0.9545 (2)	0.5220 (4)	0.6545 (4)	0.0340 (13)	
C41	0.9835 (3)	0.3542 (5)	0.7199 (4)	0.0536 (17)	
H41A	1.0258	0.3733	0.7585	0.064*	
H41B	0.9878	0.3246	0.6694	0.064*	
C42	0.9540 (3)	0.2769 (6)	0.7571 (5)	0.075 (2)	
H42A	0.9798	0.2122	0.7706	0.112*	
H42B	0.9120	0.2594	0.7185	0.112*	
H42C	0.9504	0.3070	0.8073	0.112*	
C43	0.9085 (3)	0.7272 (5)	0.4543 (4)	0.0462 (15)	
H43A	0.9490	0.7030	0.4509	0.055*	
H43B	0.8936	0.7885	0.4166	0.055*	
C44	0.8618 (3)	0.6393 (5)	0.4277 (4)	0.0432 (15)	
H44A	0.8574	0.6171	0.3720	0.065*	
H44B	0.8210	0.6641	0.4285	0.065*	
H44C	0.8761	0.5788	0.4653	0.065*	
C45	0.5806 (3)	0.5323 (4)	0.5479 (4)	0.0415 (15)	
H45A	0.5947	0.4691	0.5836	0.050*	
H45B	0.5376	0.5505	0.5458	0.050*	
C46	0.6234 (3)	0.6228 (5)	0.5839 (4)	0.0492 (16)	
H46A	0.6224	0.6387	0.6387	0.074*	
H46B	0.6095	0.6856	0.5487	0.074*	
H46C	0.6663	0.6041	0.5882	0.074*	

### Atomic displacement parameters (Å^2^)

**Table:**

	*U*^11^	*U*^22^	*U*^33^	*U*^12^	*U*^13^	*U*^23^
S1	0.0252 (6)	0.0213 (6)	0.0217 (7)	0.0029 (5)	0.0071 (5)	−0.0010 (5)
S2	0.0292 (7)	0.0309 (8)	0.0350 (8)	0.0008 (6)	0.0161 (6)	0.0001 (6)
S3	0.0247 (7)	0.0228 (7)	0.0364 (8)	−0.0001 (5)	0.0082 (6)	−0.0052 (6)
S4	0.0395 (9)	0.0491 (10)	0.0624 (12)	−0.0001 (7)	0.0233 (8)	−0.0027 (8)
O1	0.0339 (19)	0.0236 (19)	0.031 (2)	0.0047 (16)	0.0105 (16)	−0.0007 (15)
O2	0.0310 (19)	0.030 (2)	0.0183 (19)	0.0004 (15)	0.0073 (15)	−0.0036 (14)
O3	0.0292 (18)	0.0232 (18)	0.026 (2)	0.0015 (15)	0.0108 (16)	0.0008 (15)
O4	0.030 (2)	0.0216 (19)	0.054 (3)	0.0012 (16)	0.0142 (18)	−0.0043 (17)
O5	0.036 (2)	0.040 (2)	0.030 (2)	−0.0021 (17)	0.0082 (17)	−0.0061 (17)
O6	0.027 (2)	0.047 (3)	0.050 (3)	0.0079 (19)	0.0093 (19)	−0.004 (2)
O7	0.055 (3)	0.038 (2)	0.047 (3)	0.003 (2)	0.015 (2)	0.0058 (19)
O8	0.047 (2)	0.0231 (19)	0.035 (2)	0.0059 (17)	0.0223 (18)	0.0018 (16)
N1	0.023 (2)	0.036 (3)	0.035 (3)	0.001 (2)	0.005 (2)	0.006 (2)
N2	0.021 (2)	0.028 (2)	0.025 (2)	−0.0039 (18)	0.0066 (18)	0.0008 (18)
N3	0.020 (2)	0.020 (2)	0.026 (2)	0.0016 (17)	0.0077 (18)	0.0059 (17)
N4	0.020 (2)	0.024 (2)	0.024 (2)	0.0039 (18)	0.0063 (17)	0.0018 (18)
N5	0.022 (2)	0.023 (2)	0.022 (2)	0.0007 (17)	0.0063 (17)	0.0004 (17)
N6	0.020 (2)	0.026 (2)	0.046 (3)	−0.0009 (18)	0.009 (2)	−0.004 (2)
C1	0.031 (3)	0.053 (4)	0.073 (5)	−0.006 (3)	−0.004 (3)	0.017 (4)
C2	0.027 (3)	0.034 (3)	0.049 (4)	−0.004 (3)	0.009 (3)	0.002 (3)
C3	0.026 (3)	0.036 (3)	0.046 (4)	−0.008 (2)	0.008 (3)	0.000 (3)
C4	0.029 (3)	0.046 (4)	0.059 (4)	−0.014 (3)	0.004 (3)	0.002 (3)
C5	0.030 (3)	0.041 (4)	0.060 (4)	−0.010 (3)	0.009 (3)	0.013 (3)
C6	0.031 (3)	0.035 (3)	0.026 (3)	−0.006 (2)	0.012 (2)	0.003 (2)
C7	0.043 (3)	0.025 (3)	0.021 (3)	−0.006 (2)	0.014 (2)	−0.001 (2)
C8	0.035 (3)	0.031 (3)	0.032 (3)	0.001 (2)	0.014 (3)	−0.003 (2)
C9	0.027 (3)	0.035 (3)	0.038 (3)	0.005 (2)	0.014 (2)	−0.005 (2)
C10	0.025 (3)	0.028 (3)	0.030 (3)	−0.001 (2)	0.014 (2)	0.002 (2)
C11	0.025 (3)	0.028 (3)	0.027 (3)	−0.003 (2)	0.009 (2)	0.000 (2)
C12	0.028 (3)	0.028 (3)	0.028 (3)	−0.005 (2)	0.011 (2)	0.003 (2)
C13	0.018 (2)	0.028 (3)	0.028 (3)	0.000 (2)	0.008 (2)	−0.001 (2)
C14	0.028 (3)	0.038 (3)	0.017 (3)	−0.006 (2)	0.005 (2)	0.010 (2)
C15	0.028 (3)	0.035 (3)	0.023 (3)	0.001 (2)	0.005 (2)	0.003 (2)
C16	0.019 (2)	0.028 (3)	0.020 (3)	0.000 (2)	0.008 (2)	0.002 (2)
C17	0.028 (3)	0.023 (3)	0.018 (3)	0.000 (2)	0.007 (2)	0.004 (2)
C18	0.024 (2)	0.030 (3)	0.016 (3)	−0.001 (2)	0.002 (2)	0.002 (2)
C19	0.024 (2)	0.020 (3)	0.024 (3)	−0.005 (2)	0.004 (2)	−0.007 (2)
C20	0.032 (3)	0.024 (3)	0.027 (3)	0.007 (2)	0.012 (2)	0.003 (2)
C21	0.042 (3)	0.026 (3)	0.048 (4)	0.013 (3)	0.017 (3)	0.013 (3)
C22	0.033 (3)	0.025 (3)	0.029 (3)	−0.001 (2)	0.006 (2)	−0.001 (2)
C23	0.027 (3)	0.027 (3)	0.015 (2)	−0.002 (2)	0.009 (2)	−0.003 (2)
C24	0.022 (2)	0.026 (3)	0.018 (3)	−0.002 (2)	0.009 (2)	−0.005 (2)
C25	0.025 (3)	0.026 (3)	0.029 (3)	−0.003 (2)	0.008 (2)	−0.001 (2)
C26	0.038 (3)	0.025 (3)	0.023 (3)	−0.004 (2)	0.011 (2)	−0.001 (2)
C27	0.034 (3)	0.021 (3)	0.023 (3)	0.001 (2)	0.014 (2)	0.000 (2)
C28	0.047 (3)	0.017 (3)	0.028 (3)	−0.001 (2)	0.016 (3)	−0.002 (2)
C29	0.046 (3)	0.022 (3)	0.030 (3)	0.011 (3)	0.010 (3)	0.002 (2)
C30	0.030 (3)	0.031 (3)	0.024 (3)	0.004 (2)	0.005 (2)	−0.006 (2)
C31	0.036 (3)	0.018 (3)	0.033 (3)	0.004 (2)	0.012 (2)	0.002 (2)
C32	0.027 (3)	0.023 (3)	0.022 (3)	0.003 (2)	0.005 (2)	−0.008 (2)
C33	0.019 (2)	0.023 (3)	0.023 (3)	0.001 (2)	0.007 (2)	0.002 (2)
C34	0.022 (2)	0.017 (2)	0.025 (3)	0.002 (2)	0.007 (2)	0.000 (2)
C35	0.030 (3)	0.026 (3)	0.020 (3)	0.001 (2)	0.012 (2)	0.002 (2)
C36	0.025 (3)	0.024 (3)	0.025 (3)	0.004 (2)	0.010 (2)	−0.002 (2)
C37	0.023 (2)	0.018 (2)	0.027 (3)	0.005 (2)	0.009 (2)	0.002 (2)
C38	0.020 (2)	0.023 (3)	0.034 (3)	−0.003 (2)	0.012 (2)	0.006 (2)
C39	0.026 (3)	0.025 (3)	0.024 (3)	0.001 (2)	0.010 (2)	0.004 (2)
C40	0.021 (3)	0.033 (3)	0.045 (4)	−0.010 (2)	0.008 (2)	0.002 (3)
C41	0.045 (4)	0.041 (4)	0.068 (5)	0.001 (3)	0.010 (3)	0.010 (3)
C42	0.048 (4)	0.055 (5)	0.109 (7)	0.012 (4)	0.011 (4)	0.035 (4)
C43	0.046 (4)	0.055 (4)	0.043 (4)	0.010 (3)	0.021 (3)	0.011 (3)
C44	0.041 (3)	0.058 (4)	0.034 (3)	−0.004 (3)	0.017 (3)	−0.002 (3)
C45	0.066 (4)	0.034 (3)	0.037 (4)	0.002 (3)	0.033 (3)	0.000 (3)
C46	0.038 (3)	0.077 (5)	0.030 (4)	−0.005 (3)	0.008 (3)	−0.012 (3)

### Geometric parameters (Å, °)

**Table:**

S1—O1	1.427 (3)	C17—C18	1.385 (6)
S1—O2	1.432 (3)	C17—H17	0.9500
S1—N3	1.659 (4)	C18—H18	0.9500
S1—C16	1.751 (5)	C20—C21	1.498 (7)
S2—C19	1.644 (5)	C20—H20A	0.9900
S3—O4	1.419 (4)	C20—H20B	0.9900
S3—O5	1.421 (4)	C21—H21A	0.9800
S3—N6	1.653 (4)	C21—H21B	0.9800
S3—C37	1.766 (5)	C21—H21C	0.9800
S4—C40	1.650 (5)	C22—C23	1.490 (7)
O3—C19	1.332 (6)	C22—H22A	0.9800
O3—C20	1.456 (5)	C22—H22B	0.9800
O6—C40	1.335 (6)	C22—H22C	0.9800
O6—C41	1.411 (7)	C23—C24	1.401 (7)
O7—C43	1.465 (7)	C24—C33	1.372 (6)
O7—H7	0.8400	C24—C25	1.494 (6)
O8—C45	1.431 (6)	C25—C26	1.536 (7)
O8—H8	0.8400	C25—H25A	0.9900
N1—C2	1.341 (6)	C25—H25B	0.9900
N1—N2	1.361 (5)	C26—C27	1.500 (7)
N2—C12	1.366 (6)	C26—H26A	0.9900
N2—C13	1.427 (6)	C26—H26B	0.9900
N3—C19	1.359 (6)	C27—C28	1.385 (7)
N3—H3	0.8800	C27—C32	1.410 (7)
N4—C23	1.338 (6)	C28—C29	1.393 (7)
N4—N5	1.365 (5)	C28—H28	0.9500
N5—C33	1.362 (6)	C29—C30	1.399 (7)
N5—C34	1.421 (6)	C29—H29	0.9500
N6—C40	1.365 (6)	C30—C31	1.373 (7)
N6—H6	0.8800	C30—H30	0.9500
C1—C2	1.486 (7)	C31—C32	1.401 (7)
C1—H1A	0.9800	C31—H31	0.9500
C1—H1B	0.9800	C32—C33	1.458 (6)
C1—H1C	0.9800	C34—C35	1.390 (6)
C2—C3	1.405 (8)	C34—C39	1.393 (6)
C3—C12	1.380 (7)	C35—C36	1.389 (7)
C3—C4	1.507 (7)	C35—H35	0.9500
C4—C5	1.527 (8)	C36—C37	1.367 (7)
C4—H4A	0.9900	C36—H36	0.9500
C4—H4B	0.9900	C37—C38	1.396 (7)
C5—C6	1.512 (7)	C38—C39	1.376 (6)
C5—H5A	0.9900	C38—H38	0.9500
C5—H5B	0.9900	C39—H39	0.9500
C6—C7	1.385 (7)	C41—C42	1.451 (9)
C6—C11	1.410 (7)	C41—H41A	0.9900
C7—C8	1.386 (7)	C41—H41B	0.9900
C7—H7A	0.9500	C42—H42A	0.9800
C8—C9	1.380 (7)	C42—H42B	0.9800
C8—H8A	0.9500	C42—H42C	0.9800
C9—C10	1.383 (7)	C43—C44	1.487 (8)
C9—H9	0.9500	C43—H43A	0.9900
C10—C11	1.390 (6)	C43—H43B	0.9900
C10—H10	0.9500	C44—H44A	0.9800
C11—C12	1.460 (7)	C44—H44B	0.9800
C13—C14	1.381 (7)	C44—H44C	0.9800
C13—C18	1.388 (7)	C45—C46	1.483 (8)
C14—C15	1.375 (7)	C45—H45A	0.9900
C14—H14	0.9500	C45—H45B	0.9900
C15—C16	1.397 (7)	C46—H46A	0.9800
C15—H15	0.9500	C46—H46B	0.9800
C16—C17	1.385 (6)	C46—H46C	0.9800
			
O1—S1—O2	119.2 (2)	C20—C21—H21C	109.5
O1—S1—N3	103.7 (2)	H21A—C21—H21C	109.5
O2—S1—N3	110.19 (19)	H21B—C21—H21C	109.5
O1—S1—C16	109.0 (2)	C23—C22—H22A	109.5
O2—S1—C16	108.6 (2)	C23—C22—H22B	109.5
N3—S1—C16	105.2 (2)	H22A—C22—H22B	109.5
O4—S3—O5	119.6 (2)	C23—C22—H22C	109.5
O4—S3—N6	103.3 (2)	H22A—C22—H22C	109.5
O5—S3—N6	110.7 (2)	H22B—C22—H22C	109.5
O4—S3—C37	109.1 (2)	N4—C23—C24	110.4 (4)
O5—S3—C37	107.6 (2)	N4—C23—C22	120.3 (4)
N6—S3—C37	105.8 (2)	C24—C23—C22	129.3 (4)
C19—O3—C20	119.0 (4)	C33—C24—C23	106.2 (4)
C40—O6—C41	119.8 (4)	C33—C24—C25	120.6 (4)
C43—O7—H7	109.5	C23—C24—C25	133.2 (4)
C45—O8—H8	109.5	C24—C25—C26	107.9 (4)
C2—N1—N2	105.7 (4)	C24—C25—H25A	110.1
N1—N2—C12	111.6 (4)	C26—C25—H25A	110.1
N1—N2—C13	117.5 (4)	C24—C25—H25B	110.1
C12—N2—C13	130.7 (4)	C26—C25—H25B	110.1
C19—N3—S1	126.7 (3)	H25A—C25—H25B	108.4
C19—N3—H3	116.6	C27—C26—C25	112.2 (4)
S1—N3—H3	116.6	C27—C26—H26A	109.2
C23—N4—N5	105.4 (4)	C25—C26—H26A	109.2
C33—N5—N4	111.4 (4)	C27—C26—H26B	109.2
C33—N5—C34	130.6 (4)	C25—C26—H26B	109.2
N4—N5—C34	117.6 (4)	H26A—C26—H26B	107.9
C40—N6—S3	126.7 (4)	C28—C27—C32	118.8 (5)
C40—N6—H6	116.6	C28—C27—C26	121.7 (4)
S3—N6—H6	116.6	C32—C27—C26	119.5 (4)
C2—C1—H1A	109.5	C27—C28—C29	121.3 (5)
C2—C1—H1B	109.5	C27—C28—H28	119.4
H1A—C1—H1B	109.5	C29—C28—H28	119.4
C2—C1—H1C	109.5	C28—C29—C30	119.7 (5)
H1A—C1—H1C	109.5	C28—C29—H29	120.2
H1B—C1—H1C	109.5	C30—C29—H29	120.2
N1—C2—C3	110.3 (5)	C31—C30—C29	119.6 (5)
N1—C2—C1	119.7 (5)	C31—C30—H30	120.2
C3—C2—C1	129.9 (5)	C29—C30—H30	120.2
C12—C3—C2	106.1 (5)	C30—C31—C32	121.1 (5)
C12—C3—C4	121.1 (5)	C30—C31—H31	119.5
C2—C3—C4	132.7 (5)	C32—C31—H31	119.5
C3—C4—C5	107.9 (5)	C31—C32—C27	119.5 (5)
C3—C4—H4A	110.1	C31—C32—C33	124.7 (4)
C5—C4—H4A	110.1	C27—C32—C33	115.8 (4)
C3—C4—H4B	110.1	N5—C33—C24	106.5 (4)
C5—C4—H4B	110.1	N5—C33—C32	131.3 (4)
H4A—C4—H4B	108.4	C24—C33—C32	122.2 (4)
C6—C5—C4	112.8 (5)	C35—C34—C39	121.4 (4)
C6—C5—H5A	109.0	C35—C34—N5	120.6 (4)
C4—C5—H5A	109.0	C39—C34—N5	118.0 (4)
C6—C5—H5B	109.0	C36—C35—C34	118.5 (4)
C4—C5—H5B	109.0	C36—C35—H35	120.7
H5A—C5—H5B	107.8	C34—C35—H35	120.7
C7—C6—C11	119.1 (5)	C37—C36—C35	120.0 (5)
C7—C6—C5	120.5 (5)	C37—C36—H36	120.0
C11—C6—C5	120.4 (5)	C35—C36—H36	120.0
C6—C7—C8	121.0 (5)	C36—C37—C38	121.5 (4)
C6—C7—H7A	119.5	C36—C37—S3	120.8 (4)
C8—C7—H7A	119.5	C38—C37—S3	117.5 (3)
C9—C8—C7	119.6 (5)	C39—C38—C37	119.0 (4)
C9—C8—H8A	120.2	C39—C38—H38	120.5
C7—C8—H8A	120.2	C37—C38—H38	120.5
C8—C9—C10	120.5 (5)	C38—C39—C34	119.4 (5)
C8—C9—H9	119.8	C38—C39—H39	120.3
C10—C9—H9	119.8	C34—C39—H39	120.3
C9—C10—C11	120.4 (5)	O6—C40—N6	110.8 (4)
C9—C10—H10	119.8	O6—C40—S4	125.9 (4)
C11—C10—H10	119.8	N6—C40—S4	123.3 (4)
C10—C11—C6	119.3 (5)	O6—C41—C42	107.0 (5)
C10—C11—C12	125.1 (4)	O6—C41—H41A	110.3
C6—C11—C12	115.4 (4)	C42—C41—H41A	110.3
N2—C12—C3	106.3 (4)	O6—C41—H41B	110.3
N2—C12—C11	131.0 (4)	C42—C41—H41B	110.3
C3—C12—C11	122.7 (5)	H41A—C41—H41B	108.6
C14—C13—C18	121.4 (4)	C41—C42—H42A	109.5
C14—C13—N2	120.3 (4)	C41—C42—H42B	109.5
C18—C13—N2	118.3 (4)	H42A—C42—H42B	109.5
C15—C14—C13	119.6 (4)	C41—C42—H42C	109.5
C15—C14—H14	120.2	H42A—C42—H42C	109.5
C13—C14—H14	120.2	H42B—C42—H42C	109.5
C14—C15—C16	119.6 (5)	O7—C43—C44	111.9 (5)
C14—C15—H15	120.2	O7—C43—H43A	109.2
C16—C15—H15	120.2	C44—C43—H43A	109.2
C17—C16—C15	120.6 (4)	O7—C43—H43B	109.2
C17—C16—S1	120.5 (3)	C44—C43—H43B	109.2
C15—C16—S1	118.6 (4)	H43A—C43—H43B	107.9
C16—C17—C18	119.6 (4)	C43—C44—H44A	109.5
C16—C17—H17	120.2	C43—C44—H44B	109.5
C18—C17—H17	120.2	H44A—C44—H44B	109.5
C17—C18—C13	119.2 (4)	C43—C44—H44C	109.5
C17—C18—H18	120.4	H44A—C44—H44C	109.5
C13—C18—H18	120.4	H44B—C44—H44C	109.5
O3—C19—N3	110.5 (4)	O8—C45—C46	111.8 (4)
O3—C19—S2	126.4 (4)	O8—C45—H45A	109.3
N3—C19—S2	123.1 (4)	C46—C45—H45A	109.3
O3—C20—C21	105.0 (4)	O8—C45—H45B	109.3
O3—C20—H20A	110.7	C46—C45—H45B	109.3
C21—C20—H20A	110.7	H45A—C45—H45B	107.9
O3—C20—H20B	110.7	C45—C46—H46A	109.5
C21—C20—H20B	110.7	C45—C46—H46B	109.5
H20A—C20—H20B	108.8	H46A—C46—H46B	109.5
C20—C21—H21A	109.5	C45—C46—H46C	109.5
C20—C21—H21B	109.5	H46A—C46—H46C	109.5
H21A—C21—H21B	109.5	H46B—C46—H46C	109.5
			
C2—N1—N2—C12	−0.1 (6)	C20—O3—C19—N3	176.7 (4)
C2—N1—N2—C13	175.4 (4)	C20—O3—C19—S2	−3.5 (6)
O1—S1—N3—C19	179.5 (4)	S1—N3—C19—O3	−5.8 (6)
O2—S1—N3—C19	−51.8 (4)	S1—N3—C19—S2	174.4 (3)
C16—S1—N3—C19	65.1 (4)	C19—O3—C20—C21	170.9 (4)
C23—N4—N5—C33	−0.7 (5)	N5—N4—C23—C24	0.6 (5)
C23—N4—N5—C34	−175.2 (4)	N5—N4—C23—C22	−179.8 (4)
O4—S3—N6—C40	−174.6 (4)	N4—C23—C24—C33	−0.2 (5)
O5—S3—N6—C40	56.2 (5)	C22—C23—C24—C33	−179.8 (5)
C37—S3—N6—C40	−60.1 (5)	N4—C23—C24—C25	178.9 (5)
N2—N1—C2—C3	−0.3 (6)	C22—C23—C24—C25	−0.7 (9)
N2—N1—C2—C1	−179.6 (5)	C33—C24—C25—C26	33.2 (6)
N1—C2—C3—C12	0.6 (7)	C23—C24—C25—C26	−145.9 (5)
C1—C2—C3—C12	179.7 (6)	C24—C25—C26—C27	−51.6 (5)
N1—C2—C3—C4	177.9 (6)	C25—C26—C27—C28	−142.9 (5)
C1—C2—C3—C4	−3.0 (12)	C25—C26—C27—C32	37.5 (6)
C12—C3—C4—C5	−33.0 (7)	C32—C27—C28—C29	−2.1 (7)
C2—C3—C4—C5	150.1 (6)	C26—C27—C28—C29	178.3 (5)
C3—C4—C5—C6	49.6 (7)	C27—C28—C29—C30	0.2 (8)
C4—C5—C6—C7	144.4 (5)	C28—C29—C30—C31	0.8 (8)
C4—C5—C6—C11	−37.7 (8)	C29—C30—C31—C32	0.3 (8)
C11—C6—C7—C8	0.8 (8)	C30—C31—C32—C27	−2.3 (7)
C5—C6—C7—C8	178.8 (5)	C30—C31—C32—C33	−178.9 (5)
C6—C7—C8—C9	1.6 (8)	C28—C27—C32—C31	3.2 (7)
C7—C8—C9—C10	−1.9 (8)	C26—C27—C32—C31	−177.3 (4)
C8—C9—C10—C11	−0.2 (8)	C28—C27—C32—C33	−180.0 (4)
C9—C10—C11—C6	2.5 (7)	C26—C27—C32—C33	−0.4 (7)
C9—C10—C11—C12	178.1 (5)	N4—N5—C33—C24	0.6 (5)
C7—C6—C11—C10	−2.8 (7)	C34—N5—C33—C24	174.1 (4)
C5—C6—C11—C10	179.2 (5)	N4—N5—C33—C32	177.4 (5)
C7—C6—C11—C12	−178.8 (5)	C34—N5—C33—C32	−9.1 (8)
C5—C6—C11—C12	3.2 (7)	C23—C24—C33—N5	−0.2 (5)
N1—N2—C12—C3	0.5 (6)	C25—C24—C33—N5	−179.5 (4)
C13—N2—C12—C3	−174.3 (5)	C23—C24—C33—C32	−177.4 (4)
N1—N2—C12—C11	−177.2 (5)	C25—C24—C33—C32	3.3 (7)
C13—N2—C12—C11	8.1 (9)	C31—C32—C33—N5	−21.4 (8)
C2—C3—C12—N2	−0.6 (6)	C27—C32—C33—N5	161.9 (5)
C4—C3—C12—N2	−178.3 (5)	C31—C32—C33—C24	155.0 (5)
C2—C3—C12—C11	177.3 (5)	C27—C32—C33—C24	−21.7 (7)
C4—C3—C12—C11	−0.4 (8)	C33—N5—C34—C35	−41.2 (7)
C10—C11—C12—N2	18.5 (9)	N4—N5—C34—C35	132.0 (4)
C6—C11—C12—N2	−165.7 (5)	C33—N5—C34—C39	140.9 (5)
C10—C11—C12—C3	−158.8 (5)	N4—N5—C34—C39	−45.9 (6)
C6—C11—C12—C3	17.0 (7)	C39—C34—C35—C36	−1.2 (7)
N1—N2—C13—C14	−125.8 (5)	N5—C34—C35—C36	−179.0 (4)
C12—N2—C13—C14	48.6 (7)	C34—C35—C36—C37	2.6 (7)
N1—N2—C13—C18	53.1 (6)	C35—C36—C37—C38	−1.8 (7)
C12—N2—C13—C18	−132.4 (5)	C35—C36—C37—S3	173.7 (3)
C18—C13—C14—C15	0.1 (7)	O4—S3—C37—C36	−129.6 (4)
N2—C13—C14—C15	179.0 (4)	O5—S3—C37—C36	1.5 (4)
C13—C14—C15—C16	0.2 (7)	N6—S3—C37—C36	119.8 (4)
C14—C15—C16—C17	−1.5 (7)	O4—S3—C37—C38	46.0 (4)
C14—C15—C16—S1	−175.7 (4)	O5—S3—C37—C38	177.1 (3)
O1—S1—C16—C17	−52.2 (4)	N6—S3—C37—C38	−64.5 (4)
O2—S1—C16—C17	176.5 (4)	C36—C37—C38—C39	−0.6 (7)
N3—S1—C16—C17	58.5 (4)	S3—C37—C38—C39	−176.1 (4)
O1—S1—C16—C15	121.9 (4)	C37—C38—C39—C34	1.9 (7)
O2—S1—C16—C15	−9.4 (4)	C35—C34—C39—C38	−1.0 (7)
N3—S1—C16—C15	−127.3 (4)	N5—C34—C39—C38	176.8 (4)
C15—C16—C17—C18	2.6 (7)	C41—O6—C40—N6	−178.4 (5)
S1—C16—C17—C18	176.6 (4)	C41—O6—C40—S4	1.7 (8)
C16—C17—C18—C13	−2.3 (7)	S3—N6—C40—O6	−1.3 (7)
C14—C13—C18—C17	1.0 (7)	S3—N6—C40—S4	178.6 (3)
N2—C13—C18—C17	−178.0 (4)	C40—O6—C41—C42	−169.4 (6)

### Hydrogen-bond geometry (Å, °)

**Table:**

*D*—H···*A*	*D*—H	H···*A*	*D*···*A*	*D*—H···*A*
N3—H3···O8	0.88	1.82	2.700 (5)	174
N6—H6···O7	0.88	1.88	2.750 (6)	170
O7—H7···N1	0.84	2.03	2.839 (6)	161
O8—H8···N4	0.84	1.98	2.807 (5)	170
